# The activity of pomegranate extract standardized 40% ellagic acid during the healing process of incision wounds in albino rats (*Rattus norvegicus*)

**DOI:** 10.14202/vetworld.2018.321-326

**Published:** 2018-03-17

**Authors:** Wiwik Misaco Yuniarti, Hardany Primarizky, Bambang Sektiari Lukiswanto

**Affiliations:** Department of Veterinary Clinical Science, Faculty of Veterinary Medicine, Universitas Airlangga, Mulyorejo, Campus C Unair, Surabaya, 60115, Indonesia

**Keywords:** albino rats, ellagic acid, incision wound, pomegranate, wound healing process

## Abstract

**Aim:**

This research aimed to evaluate the effects of pomegranate extract standardized to 40% ellagic acid on the incised wound in albino rats.

**Materials and Methods:**

Fifty albino rats were divided into 10 treatment groups. The five groups were sacrificed on the 8^th^ day, while the others were sacrificed on the 15^th^ day. Two groups of albino rats with incised wound were not treated at all (P0), the other two groups of albino rats with incised wound were treated with Betadine^®^ (P1) ointment, and the rest of the groups were treated with pomegranate extract standardized to 40% ellagic acid with a concentration of 2.5% (P2), 5% (P3), and 7.5% (P4). The treatments were carried out twice a day with an interval of 12 h for 7 and 14 days. At the end of the research, the skin tissue of those albino rats had been taken for histopathologic preparations before H and E staining was performed.

**Results:**

Collagen deposition, polymorphonuclear neutrophils (PMN) infiltration, angiogenesis, and fibrosis degree in Group P4 treated with 7.5% pomegranate extract standardized to 40% ellagic acid for 14 days were significantly different from those in Groups P0, P1, P2, and P3, especially in the case of PMN inflammation (p<0.05).

**Conclusion:**

The administration of 7.5% pomegranate extract standardized to 40% ellagic acid for 14 days on incised wounds of those albino rats can accelerate the wound healing process characterized by collagen deposition improvement, PMN infiltration in the wound area, angiogenesis, and fibrosis degree.

## Introduction

Wound healing process is a complex phenomenon to restore the continuity of tissues and their function. Wound healing process involves several distinct and overlapping phases, namely, inflammatory phase, granulation phase, fibrogenesis phase, neovascularization phase, wound contraction phase, and epithelialization phase [1]. Effective wound management, as a result, requires an understanding of the normal wound healing process as well as is expected to be able to choose the right interventions to optimize the wound healing process [2].

Inflammation, moreover, occurs immediately after the injury, started with vasoconstriction that plays a role in triggering hemostasis process and releasing inflammatory mediators. Next, the proliferative phase is characterized by the formation of granulation tissue by fibroblasts and angiogenesis processes. Reformulation and repair of collagen fiber compartments accompanied by increased tensile strength then indicate remodeling phase [3]. However, there are several factors inhibiting wound healing process, such as recurrent trauma, poor perfusion, and oxygenation, as well as excessive inflammation [4].

Therefore, natural ingredients are considered as an essential part of health management and a good method for providing a cheap and effective health-care option, especially for wound treatment [5,6]. For instance, *Punica granatum* Linn. (Punicaceae), commonly called as pomegranate, a shrub from the Mediterranean [7,8], has been proven by several previous researches as good medicine for wound healing process [5,6,8]. Pomegranate fruit and flowers even have been widely used by the public for treating gastritis, gastrointestinal infections, gastrointestinal bleeding, dysentery, and various types of wounds [8,9].

Pomegranate, furthermore, has been known to have antitumor, antidiarrheal, antiulcer, antifungal, antioxidant, and hepatoprotector activities [7,10]. Pomegranate extract is also widely used for the treatment of diabetes mellitus and bacterial infection [11,12]. Some previous researchers even have proven that pomegranate extract contains polyphenol compounds, namely, ellagic acid, 3.3’, 4’ -tri-O-methyl ellagic acid, ethyl brevifolin carboxylate, maslinic acid, daucosterol, and tannin [13,14]. Ellagic acid has antioxidant and anti-inflammatory activity through free radical scavenging, regulation of phase 1 and 1 enzymes, modulates the secretion of proinflammatory and profibrotic cytokines and regulates the biochemical pathways that involved in lipid synthesis and degradation during inflammation [15]. Previous studies have been reported the compounds that responsible for antioxidant, anti-inflammatory, and antibacterial activities. Tannins, which consisted of punicallin, punicalagin, pedunculagin, gallic acid, and casuarinin are responsible as antioxidant. The ellagic acid, gallic acid, punicalagin, granatin, and gallagyldilactone are compounds that have anti-inflammatory effect. Catechin, epicatechin, epigallocathecin-3-gallate, flavan-3-ol, kaempferol, and quercetin are compounds that have antibacterial activity and have reported also as an antioxidant, anti-inflammatory, antiviral and anti-neoplastic [16-18]. Unfortunately, there was still a lack of researchers on the potency of pomegranate extract standardized to 40% ellagic acid for wound healing. Thus, this research aimed to reveal the effects of pomegranate extract standardized to 40% ellagic acid as a regulator of incised wound healing process in albino rats as experimental animals.

## Materials and Methods

### Ethical approval

This research has been approved by the ethical commission of Faculty of Dental Medicine, Universitas Airlangga, with Ethical Clearance Certificate’s Number 108/HRECC.FODM/VII/2017.

### Experimental animals

In this research, 50 healthy male albino rats (*Rattus norvegicus*) aged 3 months old and weighed 150-180 g were used as experimental animals.

### Research materials

Materials used in this research were whole fruit extract of pomegranate (*P. granatum* Linn.) standardized with 40% ellagic acid produced by Xi’an Bio-Technology Co., Ltd., Betadine^®^ (PT One Med), Ketamin^®^ (PT Guardian Pharmatama), diazepam, vaseline flavum, 70% ethanol, 10% formalin buffer, alcohol at various concentrations, pellet, and sterile aquadest. This research also used some materials for making preparations of histopathological examination using hematoxylin-eosin (HE) staining technique.

### Research tools

Research tools used were some surgical tools for making incised wounds and removing skin tissue, such as gloves, scissors, sterile cotton, plastic spoon, cotton bud, tweezers, clamp arteries, scalpel number 3, ruler, Hypafix, blade number 10, and razor blade. This research also used pots to place the skins that had been excised.

Besides, mortar and stamper were used in this research for making ointments. Some tools for making histopathology preparations, such as microtome and embedding set, glass object, glass cover, staining set, as well as microscope were also used.

### Treatment

Fifty male albino rats (*R. norvegicus*) were adapted first for 1 week. During the adaptation period, they were fed with sufficient food and drink. They also got several health examinations and treatments.

After the adaptation period, rats then were randomly divided into 10 treatment groups with five different treatments. Variables then observed were histopathologic images of those albino rats’ incised skin (*R. norvegicus*).

The use of pure ellagic acid (100%) for topical administration on infected wounds that infected with *Staphylococcus aureus* showed that 1% concentration gave the best results in accelerating the process of wound healing [19]. Based on that research, the topical ointment preparations, 2.5, 5, and 7.5% of pomegranate extract that standardized 40% of ellagic acid were used in this study (ellagic acid content in each dosage is 1, 2, and 3%).

Treatments administrated in this research were as follows:

**Table:** 

P0.1:	Incised wounds without any treatment and observed on day 8
P0.2:	Incised wounds without any treatment and observed on day 15
P1.1:	Incised wounds treated with Betadine^®^ and observed on day 8
P1.2:	Incised wounds treated with Betadine^®^ and observed on day 15
P2.1:	Incised wounds treated with 2.5% pomegranate extract ointment standardized to 40% ellagic acid and then observed on day 8
P2.2:	Incised wounds treated with 2.5% pomegranate extract ointment standardized to 40% ellagic acid and then observed on day 15
P3.1:	Incised wounds treated with 5% pomegranate extract ointment standardized to 40% ellagic acid and then observed on day 8
P3.2:	Incised wounds treated with 5% pomegranate extract ointment standardized to 40% ellagic acid and then observed on day 15
P4.1:	Incised wounds treated with 7.5% pomegranate extract ointment standardized to 40% ellagic acid and then observed on day 8
P4.2:	Incised wounds treated with 7.5% pomegranate extract ointment standardized to 40% ellagic acid and then observed on day 15

In all groups, except for Group P0.1 and P0.2, those rats were shaved off in their paravertebral area of 3 cm×2.5 cm to facilitate the incision process. At the time of making the wound, those rats were anesthetized in advance with a combination of ketamine and diazepam (100 mg/ml: 5 mg/ml) at a dose of 1 ml/kg of body weight [20]. The incision then was carried straight toward the caudals along 3 cm, 0.25 cm deep, and 0.01 cm wide in the area that had been shaved [21].

Next, certain treatments were given topically by applying Betadine^®^ ointment for certain groups, and pomegranate extract ointment standardized to 40% ellagic acid for some other groups with a cotton bud. Those treatments were performed twice in a day with an interval of 12 h for 7 and 14 days [22].

Afterward, skin tissue and blood of those albino rats were taken after they were sacrificed using ether per inhalation. The skin tissue was taken with a scalpel at a depth of 0.5 cm and over 1 cm from the right, left, top, and bottom sides of the incised wound to make histopathologic preparations.

### Observation

The histologic criteria for skin preparation were defined as follows:

1. Collagen was indicated with score 2 for a normal bundle of collagen, score 1 for unorganized/edema of collagen, and score 0 for amorphous one
2. Polymorphonuclear neutrophils (PMN) was indicated with score 2 for a PMN cell count of 0-10, score 1 for a PMN cell count of 11-40, and score 0 for a PMN cell count of >40
3. Angiogenesis was categorized into three degrees, namely, mild, moderate, and severe
4. Fibrosis degree was indicated by measuring the thickness of collagen bundles, divided into three, namely, mild, moderate, and severe [23].

### Statistical analysis

All data of skin histopathology examination were analyzed by a statistical test according to the data type obtained. Mean values were compared using the Kruskal–Wallis test. If treatment had led to a marked difference, it would have continued with a further statistical test to measure the differences between the groups. *Post hoc* analysis was performed using Mann–Whitney U-test. In this case, the significance level was set at 5%. As a result, which treatment gave the best result could be determined.

## Results

In this research, evaluation was performed on the histopathologic preparations that had been made on microscopic images by scoring collagen, PMN, angiogenesis, and fibrosis degree in each treatment group on days 8 and 15.

**Figure-1:**
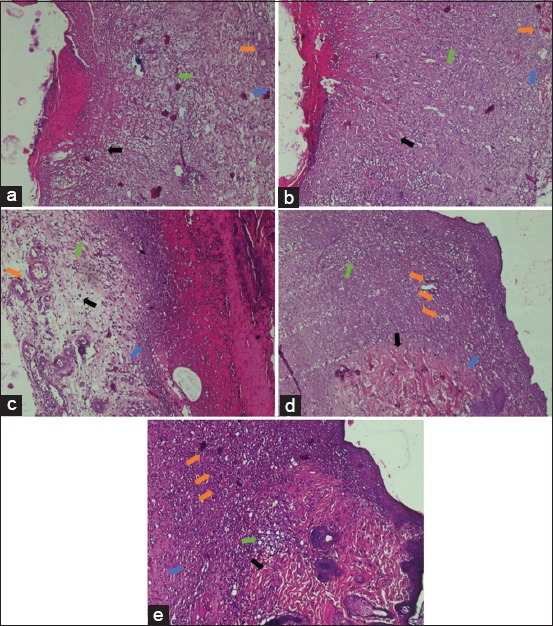
Histopathological images of those albino rats’ skin on each treatment on day 8 (H and E, 100×, [a] P0, [b] P1, [c] P2, [d] P3, [e] P4). P0 - Amorphous collagen (arrow black), PMN >40 cells (green arrow), mild angiogenesis (orange arrow), and mild fibrosis degree (blue arrow). P1 - Amorphous collagen (arrow black), PMN >40 cells (green arrow), moderate angiogenesis (orange arrow), and mild fibrosis (blue arrow). P2 - Disorganized collagen (black arrow), PMN >40 cells (green arrow), moderate angiogenesis (orange arrow), and mild fibrosis (blue arrow). P3 - Disorganized collagen (black arrow), PMN >40 cells (green arrow), moderate angiogenesis (orange arrow), and mild fibrosis (blue arrow). P4 - Disorganized collagen (black arrow), PMN >40 cells (green arrow), moderate angiogenesis (orange arrow), and moderate fibrosis (blue arrow).

**Figure-2:**
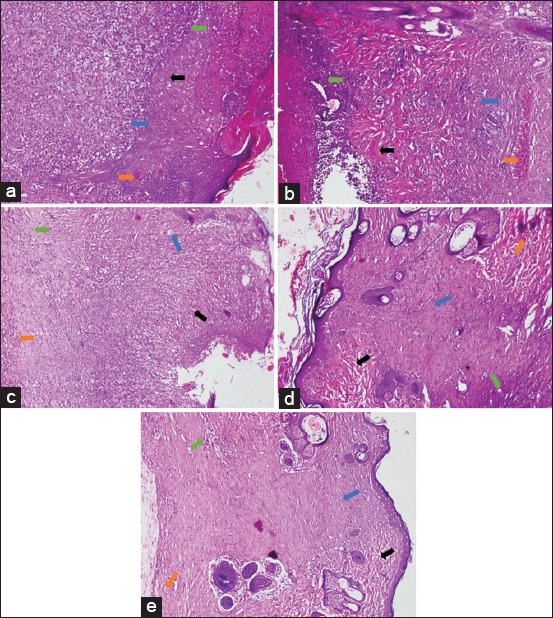
Histopathological images of those albino rat’s skin in each treatment group on day 15 (H and E, 100×, day 15 [a] P0, [b] P1, [c] P2, [d] P3, [e] P4). P1 - Disorganized collagen (black arrow), PMN >40 cells (green arrow), mild angiogenesis (orange arrow), and severe fibrosis (blue arrow). P2 - Amorphous collagen (black arrow), PMN >40 cells (green arrow), mild angiogenesis (orange arrow), and moderate fibrosis (blue arrow). P3 - Disorganized collagen (black arrow), PMN >40 cells (green arrow), mild angiogenesis (orange arrow), and severe fibrosis (blue arrow). P4 - Disorganized collagen (black arrow), PMN <40 cells (green arrow), mild angiogenesis (orange arrow), and moderate fibrosis (blue arrow).

On day 8, there was a relatively similar microscopic image in all treatment groups. The results of the evaluation showed the deposition of amorphous collagen was found in Group P0 and P1 (Figure-1a and b), while the deposition of disorganized collagen was found in Group P2, P3, and P4 (Figure-1c-e). The results also indicated that PMN cell count in all treatment groups was above 40. In addition, the degree of angiogenesis ranged from mild in Group P0 (Figure-1a) to moderate one in Group P1, P2, P3, and P4 (Figure-1b-e). Fibrosis, on the other hand, was dominated by mild degree, such as in Group P0, P1, P2, and P3 (Figure-2a-d), whereas Group P4 had moderate fibrosis (Figure-2e). Besides, the results of the statistical analysis demonstrated that there was no significant difference between Group P4 on day 8 and Groups P2 and P3 on the same day (p> 0.05). However, there was a significant difference between Group P4 on day 8 and Groups P0 and P1 (p<0.05) on the same day (Table-1).

**Table-1:** Comparison of the wound healing process on day 8.

Treatment groups	Deposition	PMN	Angiogenesis degree	Fibrosis degree
P0	0.0^a^±0.00	0.0^a^±0.00	0.8^a^±0.45	0.8^a^±0.45
P1	0.2^a^±0.45	0.0^a^±0.00	2.0^b^±0.00	1.2^ab^±0.45
P2	1.0^b^±0.00	0.2^ab^±0.45	2.2^b^±0.45	1.8^bc^±0.45
P3	0.6^ab^±0.90	0.4^ab^±0.90	1.4^ab^±0.55	2.6^c^±0.55
P4	1.4^b^±0.55	0.8^b^±0.45	2.4^b^±0.55	2.4^c^±0.45

Score values were presented in the mean±SD. Different superscripts on the same polymer showed a significant difference (p<0.05). SD=Standard deviation, PMN=Polymorphonuclear neutrophils

The results of the observation on day 15 showed a slightly different pattern. The collagen deposition found dominantly was in the disorganized form, such as in Group P1, P2, P3, and P4 (Figure-2b-e). Meanwhile, the amorphous one was only found in Group P0 (Figure-2a). The results also indicated that the PMN cell count in all treatment groups was still above 40 (Figure-2a-d), except in Group P4 (Figure-2e). Moreover, the degree of angiogenesis in all treatment groups was known to be dominated with mild degree. On the other hand, the fibrosis degree was dominated by severe ones such as in Group P0, P1, and P2 (Figure-2a-c). Meanwhile, the moderate fibrosis degree was only found in Group P1 and P4 (Figure-2d and e).

Furthermore, the results of the statistical analysis revealed that Group P4 was not significantly different from Group P2 (p>0.05). However, there was a significant difference between Group P4 and Group P0, P1, as well as P3 (p>0.05) (Table-2).

**Table-2:** Comparison of the wound healing process on day 15.

Treatment groups	Deposition	PMN	Angiogenesis degree	Fibrosis degree
P0	0.6^ab^±0.55	0.0^a^±0.00	1.0^a^±0.00	3.0^a^±0.00
P1	1.0^ab^±0.00	0.2^a^±0.45	1.6^ab^±0.55	3.0^a^±0.00
P2	0.8^ab^±0.45	0.6^ab^±0.55	1.2^ab^±0.45	2.8^a^±0.45
P3	0.4^a^±0.55	0.2^a^±0.45	1.6^ab^±0.55	3.0^a^±0.00
P4	1.6^b^±0.55	1.4^b^±0.55	1.8^b^±0.45	2.6^a^±0.55

Score values were presented in the mean±SD. Different superscripts on the same polymer showed a significant difference (p<0.05). SD=Standard deviation, PMN=Polymorphonuclear neutrophils

The provision of 7.5% pomegranate extract standardized to 40% ellagic acid for 14 days gave the best result in accelerating the healing process of incised wound in those albino rats (Table-3).

**Table-3:** Comparison of the wound healing process on day 8 and 15.

Treatment groups	Deposition		PMN		Angiogenesis degree		Fibrosis degree	
			
	Day 8	Day 15	Day 8	Day 15	Day 8	Day 15	Day 8	Day 15
P0	0.0^a^±0.00	0.6^a^±0.55	0.0^a^±0.00	0.0^a^±0.00	0.8^a^±0.45	1.0^a^±0.00	0.8^a^±0.45	3.0^b^±0.00
P1	0.2^a^±0.45	1.0^b^±0.00	0.0^a^±0.00	0.2^ab^±0.45	2.0^b^±0.00	1.6^ab^±0.55	1.2^c^±0.45	3.0^b^±0.00
P2	1.0^ab^±0.00	0.8^b^±0.45	0.2^a^±0.45	0.6^ab^±0.55	2.2^b^±0.45	1.2^a^±0.45	1.8^c^±0.45	2.8^b^±0.45
P3	0.6^ab^±0.90	0.4^a^±0.55	0.4^ab^±0.90	0.2^ab^±0.45	1.4^ab^±0.55	1.6^ab^±0.55	2.6^b^±0.55	3.0^b^±0.00
P4	1.4^ab^±0.55	1.6^b^±0.55	0.8^b^±0.45	1.4^b^±0.55	2.4^b^±0.55	1.8^b^±0.45	2.4^b^±0.45	2.6^bc^±0.55

Score values were presented in the mean±SD. Different superscripts on the same polymer showed a significant difference (p<0.05). SD=Standard deviation, PMN=Polymorphonuclear neutrophils

## Discussion

In this research, the efficacy of pomegranate extract ointment standardized to 40% ellagic acid for healing incised wounds in the albino rats was evaluated. The results showed that the observation and treatment periods can significantly influence the wound healing process characterized by collagen deposition, PMN, angiogenesis, and fibrosis degree. The results of the research indicated that 7.5% pomegranate extract ointment standardized to 40% ellagic acid could more effectively regulate the wound healing process if given 14 days than Betadine ointment as well as 2.5% and 5% pomegranate extract ointment standardized to 40% ellagic acid. Thus, in Group P4 the provision of the extract for 14 days was able to repair incised wounds with histopathologic features close to normal skin composition. The condition was characterized by well-organized and unorganized deposits, a PMN count of <40, light angiogenesis, as well as moderate fibrosis degree.

The results of the treatment scoring showed that Group P4 observed for 14 days got optimal result compared to the histopathology of normal skin since on day 14, proliferation and remodeling processes will continue for 21 days [24]. In this research, the administration of 7.5% pomegranate extract standardized to 40% ellagic acid was also known to be able to regulate the healing process by increasing collagen production, decreasing PMN cell infiltration, and increasing angiogenesis. The time required for the process is also shorter than the other treatment groups.

Pomegranates have been known to have various pharmacological activities, such as anti-inflammatory [25], antioxidant [26], antibacterial [27], antifungal [28], antispasmodic [29], antiulcer [30], and anticancer activities [31] as well as accelerate wound healing process [7]. A previous research even argues that pomegranates contain ellagic acid, tannins, and other compounds. Tannins and ellagic acids have antibactesrial activity, thus accelerating the wound healing process. Unfortunately, its mechanism is still unclear. As a result, in this research the existence of these compounds was expected can accelerate the healing process of incised wound [13]. Pure ellagic acid (100%) has antioxidant, anti-inflammatory, and antibacterial activity. When the ellagic acid 40% of pomegranate extract was used in this study, it means that there are 40% of ellagic acid and 60% of non-ellagic acid content. When the dosages used were equivalent to the most optimal dosage of pure ellagic acid (100%) are showed better results, then the strong suspicion that there is synergism of various materials that contained in pomegranate extract standardized 40%, so it also generates stronger effect [16].

In general, wound healing process can be divided into three different phases, namely, inflammatory phase, proliferation phase, and remodeling phase. Inflammation phase is characterized by the accumulation of inflammatory cells (PMN) in the wound area. The proliferative process, on the other hand, will be accompanied by epithelialization, angiogenesis, and collagen formation. Fibroblasts, collagen, edema, and new blood vessels then will soon form and get undergo maturation in the next remodeling phase. Collagen is indispensable for maintaining tissue strength in the wound area [32].

In other words, the provision of 7.5% pomegranate extracts standardized to 40% ellagic acid could accelerate the healing process of the incised wound in those rats compared to other treatment groups since pomegranate has antioxidant, anti-inflammatory, and antibacterial activities. These three activities will be synergized in the process of wound healing.

## Conclusion

The administration of 7.5% pomegranate extract standardized to 40% ellagic acid for 14 days on incised wounds of those albino rats can accelerate the wound healing process characterized by collagen deposition improvement, PMN infiltration in wound area, angiogenesis, and fibrosis degree.

## Authors’ Contributions

WMY, BSL, and HP are designed the concept for this research and scientific paper. WMY and HP have conducted the research work, e.g., maintenance of animal laboratory, collecting samples, compiling the resource materials and did the laboratory work. WMY and BSL were provided technical supports and analyzed data. All authors participated in manuscript’s draft and revision. All authors have read and approved the final manuscript.
